# Malignant Mesothelioma after Household Exposure to Asbestos

**DOI:** 10.1155/2013/570487

**Published:** 2013-10-10

**Authors:** Raya Saba, Godson Nnamdi Aronu, Ravinder Pal Bhatti, Aibek E. Mirrakhimov, Nwabundo Anusim, Aram Barbaryan, Shawn G. Kwatra, Nkemakolam Iroegbu

**Affiliations:** ^1^Saint Joseph Hospital, Department of Internal Medicine, 2900 North Lake Shore Drive, Chicago, IL 60657, USA; ^2^Department of Dermatology, Johns Hopkins University School of Medicine, Baltimore, MD, USA

## Abstract

Malignant mesothelioma (MM) is an aggressive cancer that has been closely linked to asbestos exposure. Initially recognized as an occupational cancer in male workers, MM was later found to occur in their family members as well. We report the case of an 89-year-old female who presented with abdominal distention, pain, and findings consistent with malignant ascites. Family history was significant for fatal mesothelioma in her husband of 40 years, who was a worker at a tile factory. The diagnosis of MM was confirmed on pathologic examination of the omental core biopsy.

## 1. Introduction

MM is an aggressive tumor arising from the mesothelial or submesothelial cells of the pleura, peritoneum, or pericardium. It has been recognized as an occupational cancer that is closely related to industrial asbestos exposure. Even though the latter was either restricted or forbidden many years ago, new cases of mesothelioma continue to appear because of the long latency of the disease. This has resulted in an increasing incidence of MM worldwide, a situation that is expected to continue for another 5 to 15 years [1]. 

## 2. Case

**Figure 1:**
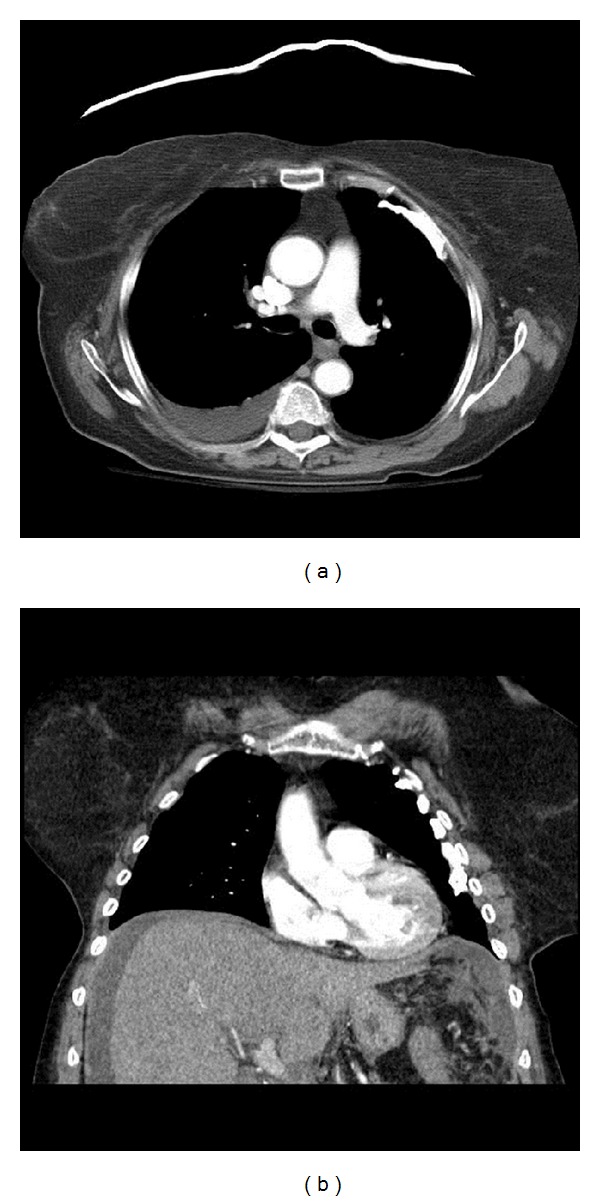
CT of the chest shows right-sided pleural effusion (a) and calcified pleural plaques in the left hemithorax (b).

**Figure 2:**
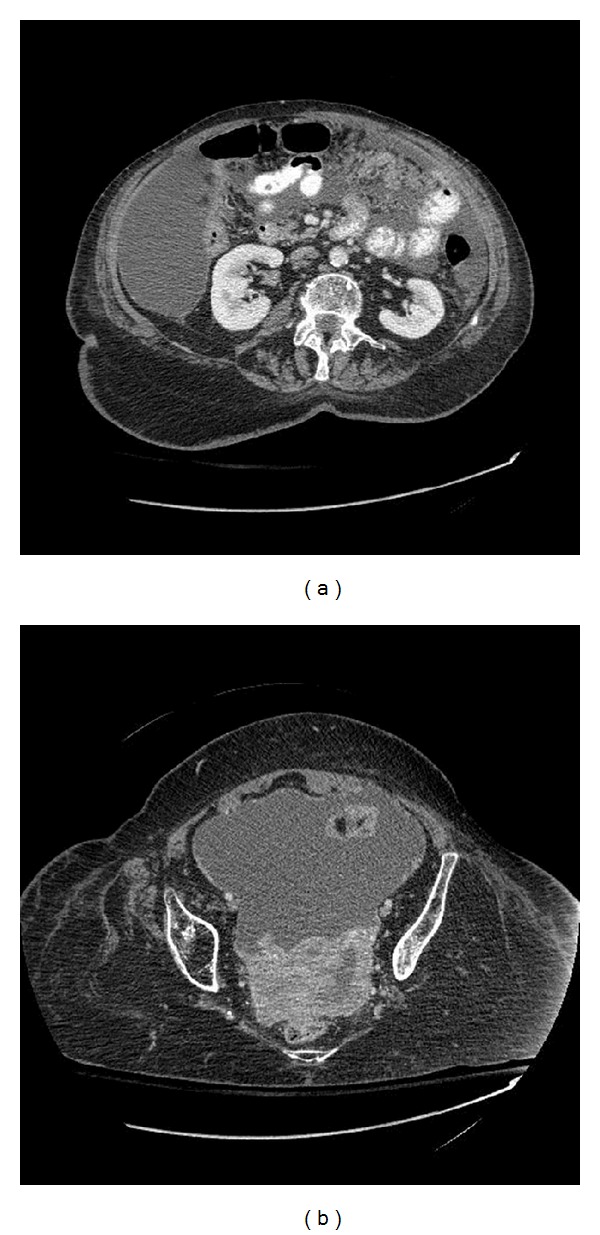
CT scan of the abdomen and pelvis shows ascites, nodularity in the peritoneal cavity (a), and prominent adnexal tissues (b).

An 89-year-old female was admitted to our hospital because of new onset abdominal pain and distention. Her symptoms had been progressing over the preceding two weeks and were associated with lower extremity edema, loss of appetite, and urinary urge incontinence. Review of systems was negative for any change in bowel habits, vaginal bleeding, or weight loss. She had a history of postpolio syndrome, hypertension, arthritis, asthma, and glaucoma. There was no history of smoking or occupational exposure to asbestos or other carcinogens. Family history was significant for colon cancer in her brother and two paternal uncles; however, the patient herself had never undergone a screening colonoscopy. Additionally, the patient's husband of 40 years had died of mesothelioma 3 years earlier. Positive findings on physical examination included pallor, a distended abdomen, paraplegia, and 1+ pitting edema in lower extremities bilaterally. Laboratory workup revealed low albumin (3.1 g/dL; normal 3.4–5.2 g/dL), mild normocytic anemia (hemoglobin 11.8 g/dL; normal 12–15.3 g/dL), and mild thrombocytosis (platelets 552 k/mm cu; normal 150–450 k/mm cu). Computed tomography (CT) of the chest, abdomen, and pelvis with contrast was done; this showed moderate to severe ascites, minimal nodularity in the peritoneal cavity suspicious for malignancy, and prominent adnexal tissues on both sides of the pelvis (Figure 2). Additionally, there was a small pleural effusion and calcified pleural plaques bilaterally (Figure 1). Tumor markers were positive for elevated CA-125 (420 U/mL; normal 0–35 U/mL). 

**Figure 3:**
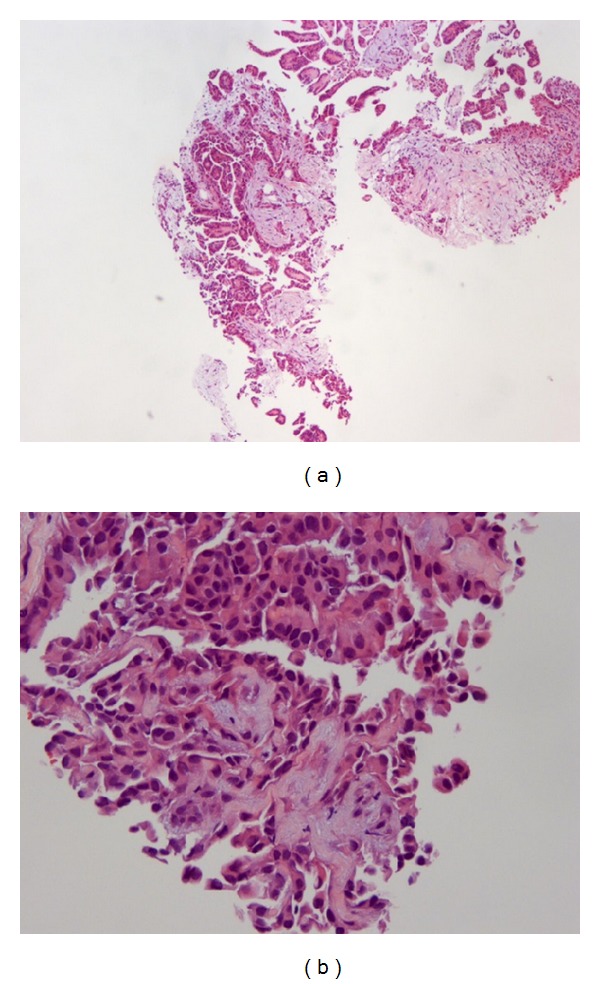
Hematoxylin and eosin (H&E) stain ((a) 5x magnification; (b) 20x magnification) showing a proliferation of abnormal mesothelial cells with moderate cellular atypia present in a papillary configuration.

Patient underwent paracentesis twice throughout her stay, for symptomatic relief. The ascitic fluid showed few atypical cells with large nuclei and prominent nucleoli. Immunohistochemistry was positive for calretinin and negative for carcinoembryonic antigen (CEA), Wilms' tumor 1 antigen (WT-1), estrogen receptor (ER), progesterone receptor (PR), and CD-15; these findings were consistent with reactive mesothelial cells as well as peritoneal mesothelioma. The patient initially did not wish to pursue any invasive diagnostic workup or treatment. However, she later agreed to an ultrasound-guided biopsy during placement of a palliative peritoneal catheter system for the management of recurrent ascites. Pathologic exam of the omental core biopsy revealed well-differentiated papillary mesothelioma of epithelioid type (Figure 3); immunohistochemistry was positive for calretinin and negative for ER, CD-15, CD19-9, and PAX8. A second pathologic review from a tertiary health care institution confirmed the same.

## 3. Discussion

### 3.1. Asbestosis and MM

Asbestos is a naturally occurring fiber that had a widespread industrial use for decades, dating back to 1858. In the early 1900s, researchers noticed a higher rate of lung disease and death in asbestos mining towns. The first convincing evidence of a link between MM and asbestos exposure was in 1960 by Wagner et al. [2], and by 1965 MM was established as a “signal tumor” of such exposure [3]. 

### 3.2. Household Exposure to Asbestos and MM

There has been an increasing body of evidence in the literature supporting the relation between MM and household exposure, with the earliest reports by Anderson in 1982 among amosite workers in Paterson, New Jersey [4]. Many years later, a study from Germany [5] reported five cases of MM in housewives, related to inhalative household contact with asbestos. This causal relationship was attributed to the cleaning of asbestos- contaminated work clothes of the husbands. In 2000, Magnani et al. [6] found an association between a moderate or high probability of domestic exposure to asbestos and an increased risk for MM after adjusting for age and sex (odds ratio (OR) 4.81, 95% confidence interval (CI) 1.8–13.1). This was related to three situations: cleaning asbestos-contaminated clothes, handling asbestos material, and presence of asbestos material susceptible to damage. This correlation was also noted by Ferrante et al. [7] in 2007. In that study, family workers were found to have a higher risk of MM, with increased standardized mortality ratio for pleural cancer of 18.00 (95% confidence interval (CI), 11.14–27.52).

### 3.3. Epidemiology

MM has a prevalence of 1-2 per million per year [8], with 80% of the cases occurring in men [9]. However, the lifetime risk of MM in exposed individuals is 4.5%–10%, which is 40 times higher than that of the average population [10]. Professionals at risk for high levels of exposure include miners, factory and ship workers, carpenters, electricians, boilermakers, insulation manufacturers, and pipe insulators [11]. 

### 3.4. Clinical Presentation

Patients with MM often present with nonspecific complaints; pleural MM frequently manifests as dyspnea and pleural pain, whereas peritoneal MM has the early symptoms of distention due to ascites, and abdominal pain. This makes MM a challenging diagnosis to establish, with a delay of up to six months prior to diagnosis [12]. Additionally, patients often present at later stages due to silent progression of the malignancy within a body cavity. This highlights the importance not only of a detailed occupational history but of a family history as well in patients with secondary or household exposure to asbestos. 

### 3.5. Diagnosis

**Figure 4:**
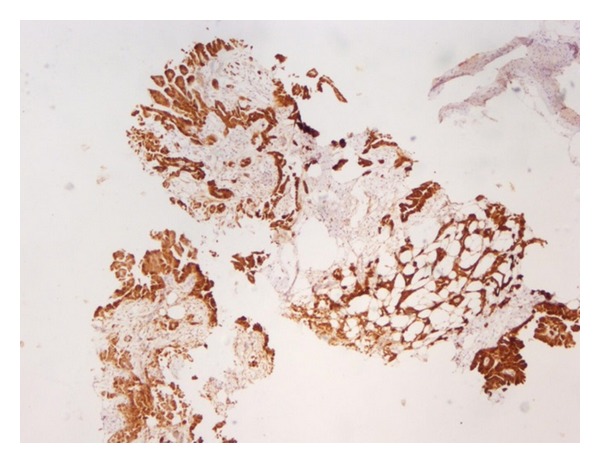
Calretinin immunohistochemical stain (5x magnification) is positive in mesothelial cells.

Cytologic analysis of the pleural or ascitic fluid may be diagnostic of MM in 33 to 84 percent of the cases [13], but a fine needle aspiration of the tumor may be needed, especially in the absence of effusion. When cytologic studies are inconclusive, a closed or opened biopsy is often indicated to make the diagnosis based on histopathological appearance. Image guidance will significantly increase the sensitivity and specificity of percutaneous needle or core biopsies. Furthermore, immunohistochemical staining plays a major role in the process (Figure 4); staining may be positive for calretinin, WT-1, cytokeratin 5/6, and epithelial membrane antigen (EMA), while negative staining may include markers consistent with other malignancies, such as CEA and desmin [14]. Assessment of the extent of tumor and metastases is completed through imaging modalities such as computed tomography (CT), magnetic resonance imaging (MRI), and positron emission tomography (PET).

### 3.6. Prognosis and Treatment

MM has a poor prognosis, with an estimated median survival ranging from 4 to 12 months [15]. Poor prognostic factors include male gender, age > 75, sarcomatoid histologic findings, low performance status (e.g., Karnofsky score) [16], and extensive disease at the time of diagnosis. Palliation with debulking surgery, pleurectomy, and decortication is appropriate in certain situations [17]. Surgery can also be performed with curative intent, in which case adjuvant chemotherapy is indicated. Due to rarity of the disease, few studies are available regarding the best chemotherapy regimen, but a combination of cisplatin and pemetrexed or gemcitabine has been shown to be effective as first line therapy [18, 19]. Other treatment modalities such as radiation and immunotherapy may be considered but remain largely experimental.

## 4. Conclusion

- MM is an aggressive tumor that has been closely related to occupational asbestos exposure.
- Household exposure of wives of workers may increase the risk of developing MM, and a high index of suspicion is warranted in such cases due to rarity of the disease.
- Clinical presentation is often nonspecific, with late detection being a common problem and ultimately contributing to the poor overall prognosis.
- Treatment modalities vary from combination of surgery and chemotherapy to palliative debulking.
